# Simultaneous stimulation of sedoheptulose 1,7‐bisphosphatase, fructose 1,6‐bisphophate aldolase and the photorespiratory glycine decarboxylase‐H protein increases CO_2_ assimilation, vegetative biomass and seed yield in Arabidopsis

**DOI:** 10.1111/pbi.12676

**Published:** 2017-03-21

**Authors:** Andrew J. Simkin, Patricia E. Lopez‐Calcagno, Philip A. Davey, Lauren R. Headland, Tracy Lawson, Stefan Timm, Hermann Bauwe, Christine A. Raines

**Affiliations:** ^1^ School of Biological Sciences University of Essex Colchester UK; ^2^ Plant Physiology Department University of Rostock Rostock Germany

**Keywords:** chlorophyll fluorescence imaging, FBP aldolase, glycine decarboxylase‐H protein, photosynthesis, SBPase, transgenic

## Abstract

In this article, we have altered the levels of three different enzymes involved in the Calvin–Benson cycle and photorespiratory pathway. We have generated transgenic Arabidopsis plants with altered combinations of sedoheptulose 1,7‐bisphosphatase (SBPase), fructose 1,6‐bisphophate aldolase (FBPA) and the glycine decarboxylase‐H protein (GDC‐H) gene identified as targets to improve photosynthesis based on previous studies. Here, we show that increasing the levels of the three corresponding proteins, either independently or in combination, significantly increases the quantum efficiency of PSII. Furthermore, photosynthetic measurements demonstrated an increase in the maximum efficiency of CO_2_ fixation in lines over‐expressing SBPase and FBPA. Moreover, the co‐expression of GDC‐H with SBPase and FBPA resulted in a cumulative positive impact on leaf area and biomass. Finally, further analysis of transgenic lines revealed a cumulative increase of seed yield in SFH lines grown in high light. These results demonstrate the potential of multigene stacking for improving the productivity of food and energy crops.

## Introduction

The accumulated photosynthate produced over the season determines the yield of a crop, but improvements in photosynthesis have not been used in traditional breeding approaches to identify high‐yielding varieties. The reasons for this are twofold, (i) methodologies to make accurate field measurements have only been available in the last 10–20 years, and also, (ii) there is a lack of evidence to determine whether there is a correlation between the rate of photosynthesis on a leaf area basis and final yield of the crop (Evans, 2013; Fischer *et al*., 1998; Gifford and Evans, 1981). There is now an urgent need to increase crop productivity and yields to meet the nutritional demands of a growing world population, and there is growing evidence that this may be achieved through improvement of photosynthetic energy conversion to biomass (von Caemmerer and Evans, 2010; Ding *et al*., 2016; Lefebvre *et al*., 2005; Long *et al*., 2006, 2015; Simkin *et al*., 2015). Evidence from a combination of theoretical studies and transgenic approaches has provided compelling evidence that manipulation of the Calvin–Benson (CB) cycle can improve energy conversion efficiency and lead to an increase in yield potential (Long *et al*., 2006; Poolman *et al*., 2000; Raines, 2003, 2006, 2011; Zhu *et al*., 2007, 2010).

Previous studies have demonstrated that even small reductions in individual CB cycle enzymes such as sedoheptulose 1,7‐bisphosphatase (SBPase) and fructose 1,6‐bisphosphate aldolase (FBPA) negatively impact on carbon assimilation and growth, indicating that these enzymes exercise significant control over photosynthetic efficiency (Ding *et al*., 2016; Haake *et al*., 1998, 1999; Harrison *et al*., 1998, 2001; Lawson *et al*., 2006; Raines, 2003; Raines and Paul, 2006; Raines *et al*., 1999). Furthermore, the disruption of the chloroplastic fructose‐1,6‐bisphosphatase (FBPase) gene was also shown to impact negatively on carbon fixation (Kossmann *et al*., 1994; Rojas‐González *et al*., 2015; Sahrawy *et al*., 2004). These results strongly suggested that improvements in photosynthetic carbon fixation may be achieved by increasing the activity of individual CB cycle enzymes. Evidence supporting this hypothesis came from transgenic tobacco plants over‐expressing SBPase (Lefebvre *et al*., 2005), the cyanobacterial bifunctional SBPase/FBPase (Miyagawa *et al*., 2001) or FBPA (Uematsu *et al*., 2012). These single manipulations resulted in increase in photosynthetic carbon assimilation, enhanced growth and an increase in biomass. More recently, Simkin *et al*. (2015) demonstrated that the combined over‐expression of SBPase and FBPA in tobacco resulted in a cumulative increase in biomass and that these increases could be further en`hanced by the over‐expression of the cyanobacterial inorganic carbon transporter B (ictB), proposed to be involved in CO_2_ transport, although its function was not established in these plants (Simkin *et al*., 2015). These results demonstrate the potential for the manipulation of photosynthesis, using multigene stacking approaches, to increase biomass yield (Simkin *et al*., 2015).

The efficiency of CO_2_ fixation by the CB cycle is compromised by the oxygenase activity of ribulose‐1,5‐bisphosphate carboxylase/oxygenase (Rubisco) which directly competes with CO_2_ fixation at the active site, resulting in the formation of 2‐phosphoglycolate (2PG) and subsequently significant energy costs and CO_2_ losses in the photorespiratory pathway, resulting in significant losses in yield (Bowes *et al*., 1971; Tolbert, 1997; Walker *et al*., 2015, 2016). Therefore, a major target to improve photosynthesis has been to reduce photorespiration, either through protein engineering to improve Rubisco catalysis or by limiting the flux through this pathway, none of which have as yet yielded positive results due to both the complexity of the Rubisco catalytic and assembly processes (Cai *et al*., 2014; Carmo‐Silva *et al*., 2015; Lin *et al*., 2014a; Orr *et al*., 2016; Sharwood *et al*., 2016; Whitney *et al*., 2011). More ambitious approaches to this problem are now being taken, including the introduction of cyanobacterial or algal CO_2_‐concentrating mechanisms, novel synthetic metabolic pathways and the introduction of the C4 pathway into C3 crops (Betti *et al*., 2016; Lin *et al.,* 2014b; McGrath and Long, 2014; Meyer *et al*., 2016; Montgomery *et al*., 2016). However, to date the only successful approach to limiting photorespiration which has resulted in an improvement in photosynthesis has been through the introduction of alternative routes to metabolize 2PG and return CO_2_ for use in the CB cycle (Dalal *et al*., 2015; Kebeish *et al*., 2007; Maier *et al*., 2012; Nolke *et al*., 2014; Peterhänsel *et al*., 2013; Xin *et al*., 2015). Reductions in the flux through the photorespiratory cycle by targeted knock‐down of GDC‐P in potato and GDC‐H in rice have been shown to lead to reductions in photosynthesis and growth rates (Engel *et al*., 2007; Heineke *et al*., 2001; Lin *et al*., 2016). The opposite approach, namely over‐expression of the glycine decarboxylase (GDC)‐H protein (GDC‐H) and glycine decarboxylase (GDC)‐L protein (GDC‐L) in *Arabidopsis thaliana* (Arabidopsis), resulted in an improvement of photosynthesis and increased vegetative biomass when compared to wild‐type plants (Timm *et al*., 2012, 2015, 2016). Although the underlying mechanism responsible for this effect has not been fully elucidated, these authors proposed that stimulation of the CB cycle is brought about by the increase in GDC activity, resulting in a reduction in the steady‐state levels of photorespiratory metabolites that may negatively impact on the function of the CB cycle (e.g. 2PG, glycolate, glyoxylate or glycine (Anderson, 1971; Kelly and Latzko, 1976; Eisenhut *et al*., 2007; Lu *et al*., 2014; Timm *et al*., 2015, 2016)).

In the light of the results from Timm *et al*. (2012, 2015), the aim of this study was to explore the possibility that the simultaneous increase in the activity of enzymes of both the CB cycle and the photorespiratory pathway could lead to a cumulative positive impact on photosynthetic carbon assimilation and yield. To test this, we have taken a proof‐of‐concept approach using the model plant Arabidopsis in which we have over expressed SBPase, FBPA and GDC‐H either alone or in combination. We have shown that the simultaneous manipulation of multiple targets can lead to a cumulative impact on biomass yield under both low‐ and high‐light growing conditions. Interestingly, we have also shown that manipulation of the photorespiratory pathway alone resulted in an increase in vegetation biomass but not seed yield. In contrast, manipulation of both the CB cycle and photorespiratory pathway increased both biomass and seed yield.

## Results

### Production and selection of arabidopsis transformants

The full‐length Arabidopsis SBPase (*At3 g55800*) and FBPA cDNA (*At4 g38970*) were used to generate three over‐expression constructs PTS1‐SB, PTS1‐FB and PTS1‐SBFB in vector pGWPTS1 (Figure S1). These transgenes were under the control of the photosynthetic tissue‐specific (PTS) *rbcS2B* (1150 bp; *At5 g38420*) promoter. These constructs were transformed into Arabidopsis using the floral dip method (Clough and Bent, 1998), and the resulting transgenic plants were selected on kanamycin‐/hygromycin‐containing medium. T2 plants expressing the integrated transgenes were screened by immunoblotting and allowed to self‐fertilize to generate seeds for T3 plants. Following initial characterisation of primary independent lines generated, 3–4 independent lines overexpressing either SBPase (S3, S8, S12) or FBPA (F6, F9, F11) and SBPase and FBPA together (SF4, SF6, SF7, SF12) were selected for further study.

**Figure 1:**
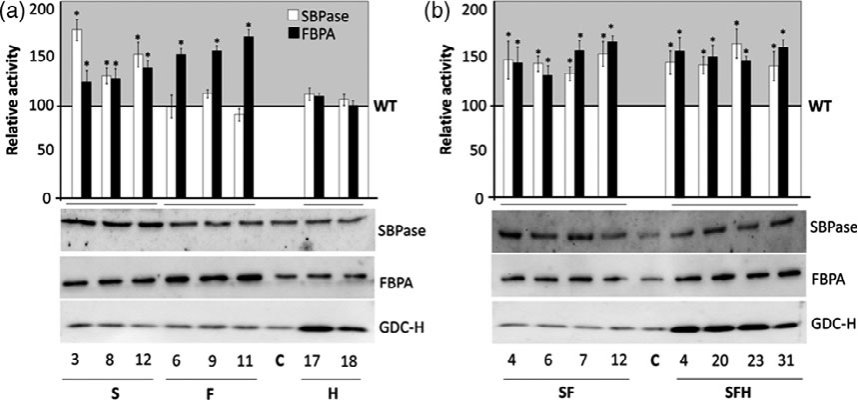
Molecular and biochemical analysis of the transgenic plants over‐expressing SBPase (S), FBPA (F) and GDC‐H (H). SBPase and FBPA enzyme activity (SBPase, FBPA) and immunoblot blot analysis (SBPase, FBPA, GDC‐H) of protein extracts from two independent leaves of (a) S, F and H lines and (b) SF and SFH lines used in this study compared to non‐transformed control (C). Enzyme assays represent data from 12 to 24 independent plants per group compared to 12–16 C plants. The results are represented as a percentage (%) of total activity for SBPase (6.7 μmol/m^2^/s) and FBP aldolase (22 μmol/m^2^/s) determined in wild type (WT). Enzyme activities per plant can be seen in Figure S2. Columns represent mean values, and standard errors are displayed. Lines that are significantly different to C are indicated (*P < 0.05).

Further analysis was carried out on T3 plants grown at 130 μmol/m^2^/s in an 8‐h/16‐h light/dark cycle and total extractable SBPase and FBPA activity determined in extracts from newly fully expanded leaves. The results are represented as a percentage (%) of total activity for SBPase (6.7 μmol/m^2^/s) and FBP aldolase (22 μmol/m^2^/s) determined in wild type (WT). This analysis showed that these plants had increased levels of SBPase (137%–185%) and FBPA (146%–180%) activity (Figure 1) compared to WT and nontransformed azygous (A) controls (azygous‐control plants used in this study were recovered from the segregating population and verified by PCR). Interestingly, a small increase in endogenous FBPA activity (125%–136%) was also observed in SBPase over‐expressing lines (Figure 1a), but no significant increase in SBPase activity was observed in lines over‐expressing FBPA.

Plants over‐expressing SBPase (S), FBPA (F) and the GDC‐H protein (H) were generated by crossing two SBPase + FBPA (SF) lines (SF6 and SF12) with two *Flaveria pringlei* GDC‐H protein (Kopriva and Bauwe, 1995) over‐expressing lines (*Fp*HL17 and *Fp*HL18) originally generated by Timm *et al*. (2012) under the control of the leaf‐specific and light‐regulated *Solanum tuberosum ST‐*LS1 promoter (Stockhaus *et al*., 1989). Four independent lines (SFH4, SFH20, SFH23 and SFH31) over‐expressing SBPase, FBPA and GDC‐H (SFH) were identified by PCR and SBPase and FBPA enzyme activities. SBPase and FBPA protein levels were found to be similar to those observed in SF lines (Figure 1b). No significant difference in SBPase or FBPA activities was observed in lines over‐expressing GDC‐H alone compared to WT/A controls (C). The full set of assays showing the variation between plants for both SBPase and FBPA activities can be seen in Figure S2.

**Figure 2:**
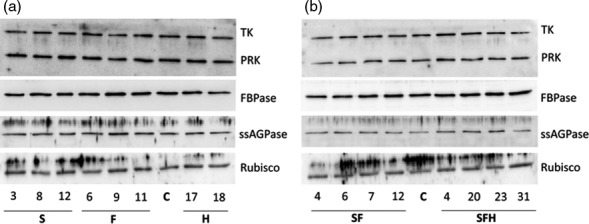
Molecular and biochemical analysis of the transgenic plants over‐expressing SBPase (S), FBPA (F) and GDC‐H (H). Immunoblot blot analysis of protein extracts from two independent leaves of (a) S, F and H lines and (b) SF and SFH lines used in this study compared to C. Transketolase (TK), phosphoribulokinase (PRK), fructose‐1,6‐bisphosphase (FBPase) the small subunit of ADP glucose pyrophosphoryalse (ssAGPase) and Rubisco.

In addition to total extractable enzyme activity, immunoblot analysis of the T3 progenies of S, F, SF, H and SFH lines was carried out using WT/A as controls (C). This analysis identified a number of plants over‐expressing SBPase or FBPA and others with increased levels of both SBPase and FBPA (Figures 1a,b and S3). Interestingly, the over‐expression of SBPase in Arabidopsis led to an increase in endogenous FBPA protein levels (Figure 1a) in agreement with the observed increase in enzyme activity. The original H lines and the newly generated SFH plants were shown to accumulate GDC‐H when compared to both nontransformed control plants and other transgenic lines (Figure 1a,b). Given the change in FBPA protein levels in the SBPase over‐expressing line, we used immunoblot analysis to determine whether there were any changes in other CB cycle enzymes. No detectable changes in the levels of transketolase (TK), phosphoribulokinase (PRK), fructose‐1,6‐bisphosphatase (FBPase), Rubisco or the ADP glucose pyrophosphorylase (ssAGPase) small protein were observed when compared to levels in C plants (Figure 2).

### Chlorophyll fluorescence imaging reveals increased photosynthetic efficiency in young over‐expressing seedlings

**Figure 3:**
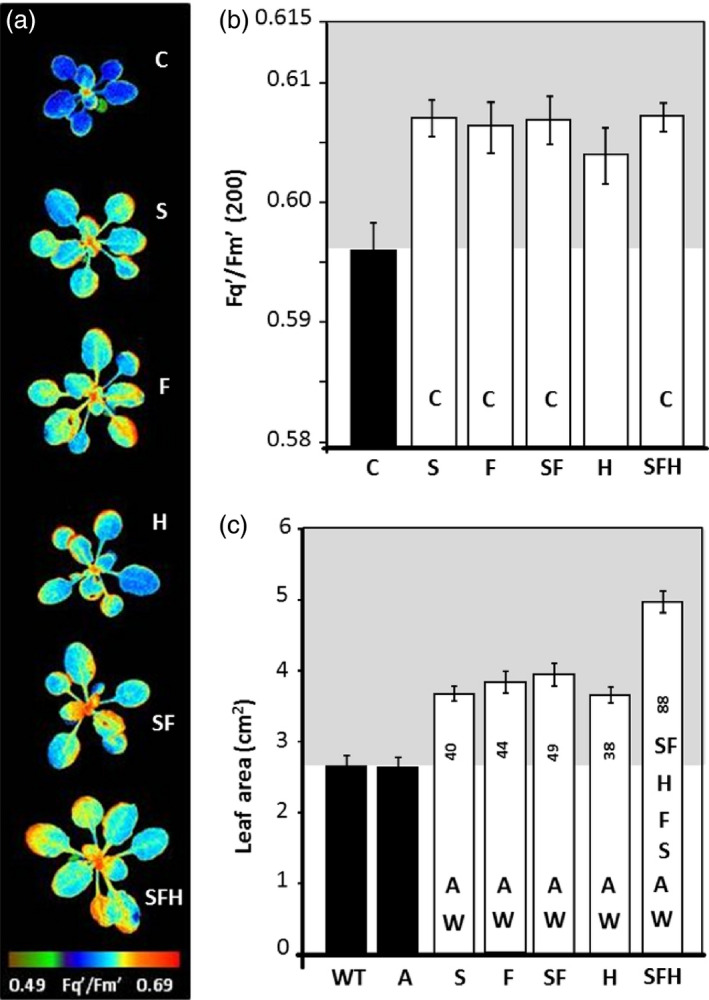
Photosynthetic capacity and leaf area in transgenic seedlings determined using chlorophyll fluorescence imaging. C and transgenic plants were grown in controlled environment conditions with a light intensity 130 μmol/m^2^/s, 8‐h light/16‐h dark cycle for 15 days and chlorophyll fluorescence imaging used to determine *F*_q_'/*F*_m_' (maximum PSII operating efficiency) values of the whole plant at (a, b) 200 μmol/m^2^/s and (c) leaf area at time of analysis. Azygous controls (A) recovered from a segregating population. Lines over‐expressing SBPase (S), FBPA (F), GDC‐H protein (H), SBPase and FBPA (SF) and SBPase, FBPA and GDC‐H (SFH) are represented. The data were obtained using six individual plants from two (H), three (S, F, SF) or four (SFH) independent transgenic lines (18–24 plants total) compared to 12 C. Columns represent mean values, and standard errors are displayed. Significant differences between lines (*P* < 0.05) are represented as capital letters indicating whether each specific line is significantly different from another (i.e. SBPase lines (S) are significantly bigger than wild type (WT) and azygous lines (A)). Numbers indicate % increases over WT.

To explore the impact of increased levels of SBPase, FBPA and the GDC‐H protein on photosynthesis, plants were grown at 130 μmol/m^2^/s in an 8‐h/16‐h light/dark cycle and the quantum efficiency of PSII photochemistry (*F*_q_'/*F*_m_') analysed using chlorophyll a fluorescence imaging (Baker, 2008; Murchie and Lawson, 2013). Plants over‐expressing SBPase and FBPA, either independently or in combination (including with GDC‐H), had a significantly higher *F*_q_'/*F*_m_' at an irradiance of 200 μmol/m^2^/s when compared to C plants (Figure 3a,b). Plants over‐expressing GDC‐H alone showed a small increase in the average levels of *F*_q_'/*F*_m_' compared to C (*P* = 0.061). When measurements were made at a higher light level (600 μmol/m^2^/s), all lines analysed, with the exception of SFH, showed a significant increase in *F*_q_'/*F*_m_' compared to C plants (Figure S4a). From images taken as part of the chlorophyll fluorescence analysis, leaf area was determined and shown to be significantly larger for all transgenic lines compared with WT and azygous (A) controls (Figure 3c). Interestingly, SFH plants showed the greatest leaf area in all experiments. No significant differences in leaf area were observed between WT and A. From this point on, C plants used were the combined data from WT and A plants.

### Photosynthetic CO_2_ assimilation rates are increased in mature plants grown in low light

**Figure 4:**
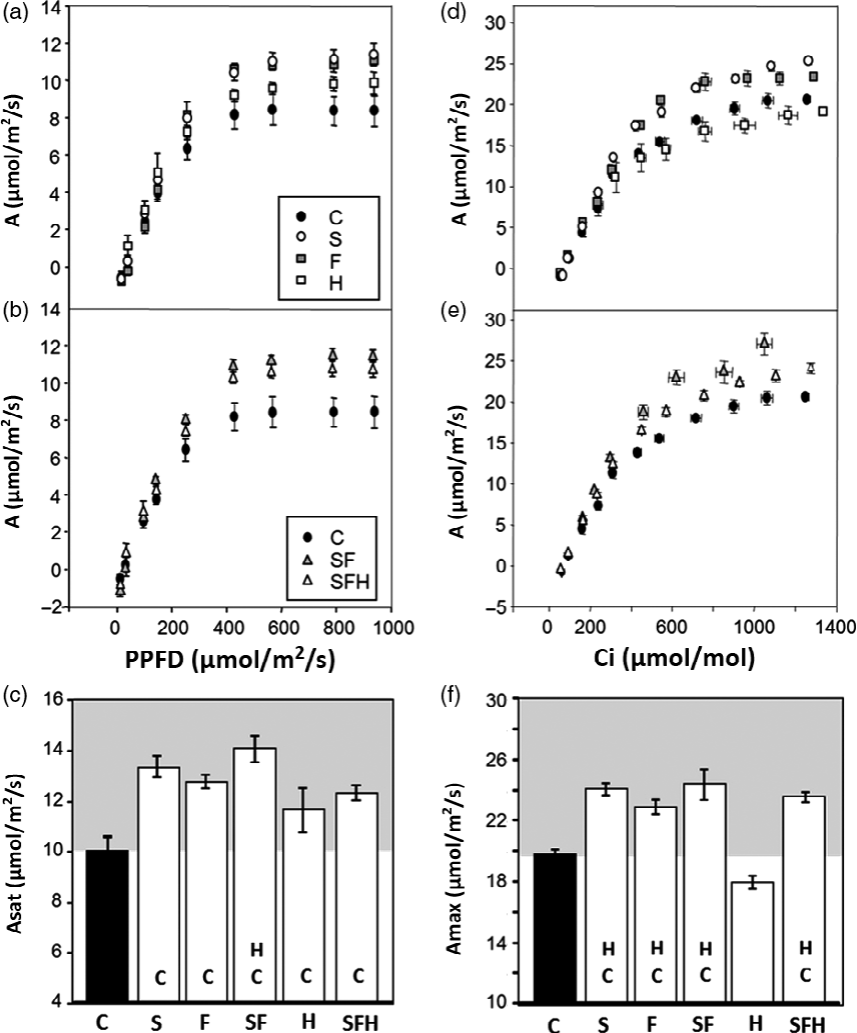
Photosynthetic responses of C and transgenic plants. (a, b) Photosynthesis carbon fixation rates were determined as a function of increasing light intensity. (c) *A*_sat_ determined from light response curves. (d, e) Photosynthetic carbon fixation rates were determined as a function of increasing CO_2_ concentrations (A/C_i_) at saturating‐light levels (1000 μmol/m^2^/s). (f) *A*_max_ determined from A/C_i_ response curves. C and transgenic plants were grown in controlled environment conditions with a light intensity 130 μmol/m^2^/s, 8‐h light/16‐h dark cycle for 4 weeks. Lines over‐expressing SBPase (S), FBPA (F), GDC‐H protein (H), SBPase and FBPA (SF) and SBPase, FBPA and GDC‐H (SFH) are represented. Columns represent mean values, and standard errors are displayed. Significant differences between lines (*P* < 0.05) are represented as capital letters indicating whether each specific line is significantly different from another. Results are based on 4–7 plants per line. (i.e. SBPase lines (S) are significantly different to controls (C)).

To explore the impact of changes in the levels of enzymes in both the CB cycle and photorespiratory pathway, CO_2_ assimilation rates were determined as a function of light intensity (Figure 4a,b). From these light response curves, the maximum light‐saturated rate of photosynthesis (*A*_sat_) was shown to be significantly higher in all transgenic plants when compared to C plants (Figure 4c). Small differences in CO_2_ assimilation rates (*A*) were also observed in the S, F, SF and SFH plants even at light intensities as low as 150 μmol/m^2^/s, which is close to that of the growth conditions (Figure S5).

We also determined *A* as a function of internal CO_2_ concentration (*C*_i_) in the same plants (Figure 4d,e). In all transgenic plants, except those over‐expressing GDC‐H alone, *A* was significantly greater at *C*_i_ concentrations above 400 μmol/mol than in C plants (Figure 4d,e). Although *A* in SFH plants was higher than in the control plants at 400 μmol/mol, it was lower than that observed in the S, F or SF plants. The maximum rate of CO_2_ assimilation (*A*_max_) was significantly higher in lines S, F, SF and SFH compared to C; however, no significant differences were observed between these lines (Figure 4f). Interestingly, the H plants show no increase in *A*_max_ when compared to C plants. Further analysis of the *A*/*C*_i_ curves using the equations published by von Caemmerer and Farquhar (1981) illustrated that the maximum rate of carboxylation by Rubisco (*Vc*_max:_ Figure S4b) in lines S, SF and SFH was significantly greater than in C, and *Vc*_max_ in these lines was also significantly greater than in lines expressing GDC‐H alone. Maximum electron transport rates (*J*_max_: Figure S4c) were also elevated in lines S, F, SF and SFH compared to C and were also shown to be significantly elevated compared to H.

**Figure 5:**
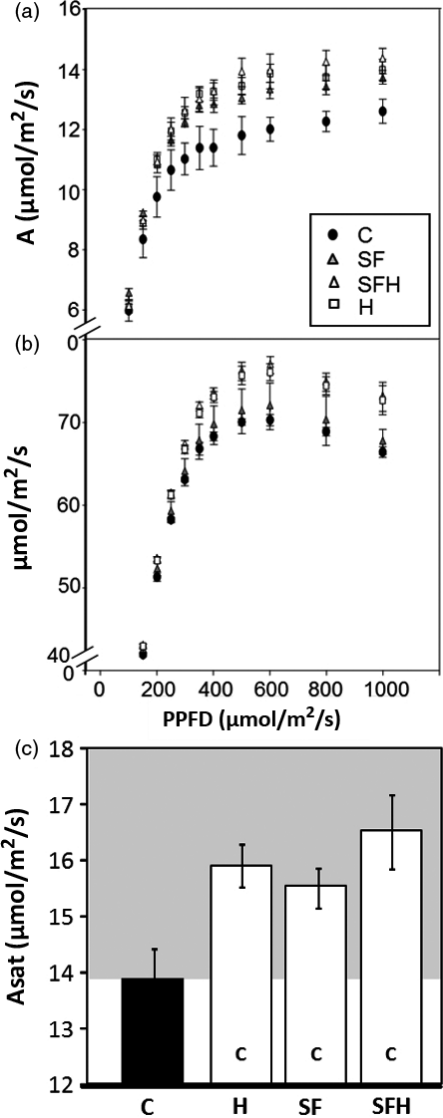
Photosynthetic responses of the transgenic plants at 2% [O_2_] (a) and (b) chloroplast electron transport rates in transgenic plants at 2% [O_2_]. (c) Mean values of *A*_sat_ determined from light response curves. C and transgenic plants were grown in controlled environment conditions with a light intensity 130 μmol/m^2^/s, 8‐h light/16‐h dark cycle for 4 weeks. Lines over‐expressing the GDC‐H protein (H), SBPase and FBPA (SF) and SBPase, FBPA and GDC‐H (SFH) are represented. Columns represent mean values, and standard errors are displayed. Significant differences between lines (*P* < 0.05) are represented as capital letters. Results are based on 5–6 plants per line compared to six controls.

To further assess the effect of the manipulation of the CB cycle and/or the GDC‐H protein, the rates of photosynthetic carbon assimilation and electron transport were determined in mature plants as a function of light intensity at 2% [O_2_] to eliminate photorespiration (Figure 5a). Electron transport rates through PSII in H and SFH over‐expression plants were significantly greater than in the C and SF plants at light levels above 300 μmol/m^2^/s (Figure 5b). *A*_sat_ was also significantly higher, 12%–19%, in all lines compared to C although no significant differences were observed between the different transgenic lines (Figure 5c).

### Increased SBPase and FBPA activity and over‐expression of the glycine decarboxylase‐H protein stimulates growth in low light

**Figure 6:**
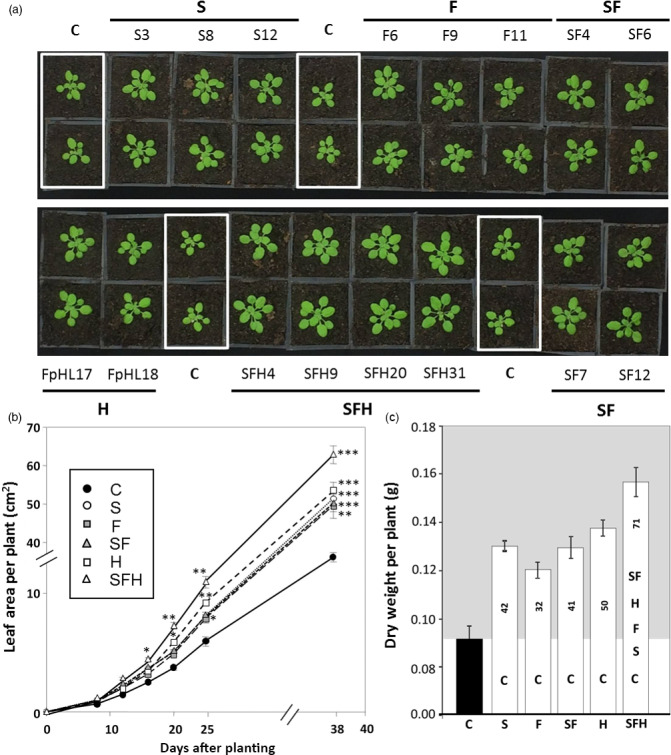
Growth analysis of C and transgenic lines grown in low light. (a) Plants were grown at 130 μmol/m^2^/s light intensity in short days (8 h/16 h days) for 15 days. (b) Plant growth rate evaluated over the first 38 days. Significant differences *(*P* < 0.05), **(*P* < 0.01), ***(*P* < 0.001) are indicated. (c) Final dry weight (g) after 38 days of development and statistical differences between lines. % increases over C are indicated within the columns. Lines over‐expressing SBPase (S), FBPA (F), GDC‐H protein (H), SBPase and FBPA (SF) and SBPase, FBPA and GDC‐H (SFH) are represented. Columns represent mean values, and standard errors are displayed. Significant differences between lines (*P* < 0.03) are represented as capital letters indicating whether each specific line is significantly different from another. Results are representative of 9–12 plants from two (H), three (S, F, SF) or four (SFH) independent lines (C plants including wild type and azygous lines segregated from primary transformants).

The growth of the different transgenic and C plants was determined using image analysis of total leaf area over a period of 38 days from planting (Figure 6a), which showed all transgenic lines had a significantly greater leaf area than C, as early as 16 days after planting (Figure 6b). Furthermore, plants over‐expressing all three transgenes (SFH) were shown to have a significantly larger leaf area when compared to all other transgenic lines including G and SF, indicating a cumulative advantage from combining these transgenes at this stage in development. This growth trend continued through to 15 days postplanting (Figure S6a). By 20 days after planting (Figure S6b), plants over‐expressing the glycine decarboxylase‐H protein (H) were shown to be significantly bigger than S, F and SF at the same time point, and triple over‐expressing lines SFH remained significantly bigger than all other lines studied (Figure 6b).

Plants were allowed to continue growing until harvest at 38 days (Figure S7). At this stage of development, no significant difference in leaf area or dry weight could be observed between S, F, H or SF lines when compared to each other (Figure 6c). However, all lines attained a significantly larger leaf area and dry weight when compared to C. Notably, at this stage, the triple over‐expressing lines SFH were significantly larger with a higher dry weight (+70%) than all other transgenic and C plants. Furthermore, lines SF and SFH both showed a significant increase in leaf number after 38 days (Figure S8).

### Increased SBPase and FBPA activity and expression of the glycine decarboxylase‐H protein impacts on the carbohydrate profile of selected lines

**Figure 7:**
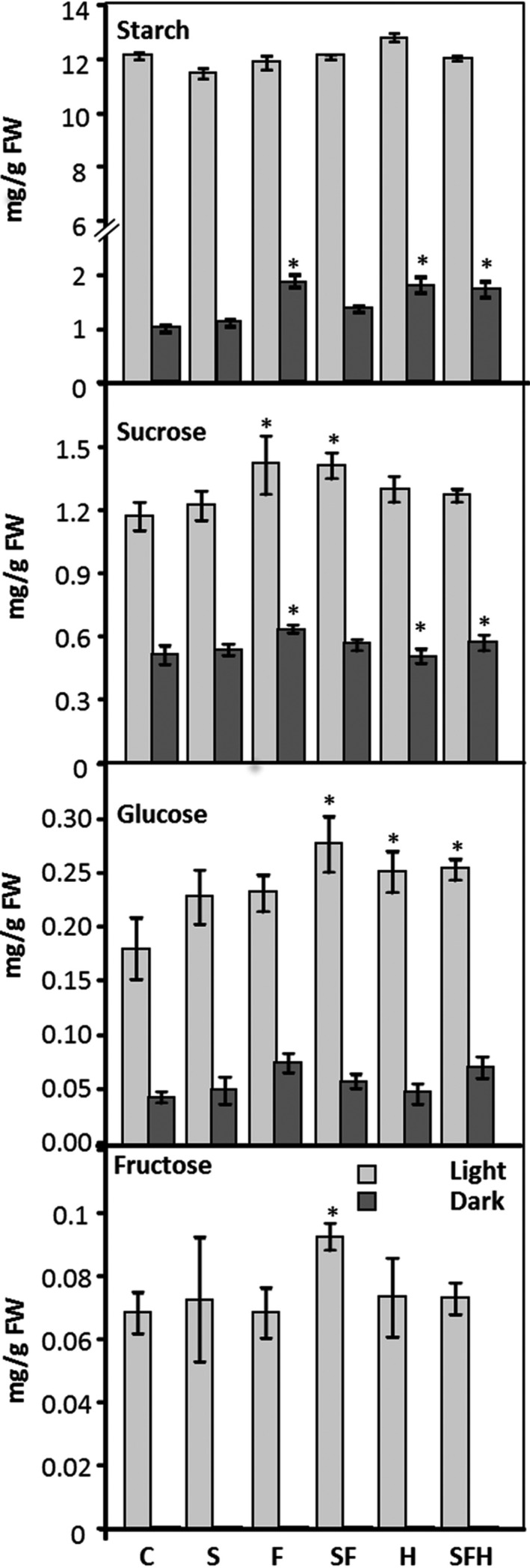
Leaf starch and sugar content at end of 8‐h light period (light grey) and end of 16‐h dark period (dark grey). Results are mean values based on 12–18 individual plants from two (H), three (S, F, SF) or four (SFH) independent transgenic lines. Columns represent mean values, and standard errors are displayed. Lines over‐expressing SBPase (S), FBPA (F), GDC‐H protein (H), SBPase and FBPA (SF) and SBPase, FBPA and GDC‐H (SFH) are represented. Significant differences between C and over‐expressing lines (*P < 0.01) are represented.

To determine how the over‐expression of these key proteins impacts on downstream processes, leaf tissue was harvested and starch and sugar content were evaluated. No significant difference in starch levels were detected at the end of the day in any of the transgenic lines compared to C (Figure 7). Interestingly, slightly higher starch levels were detected 1 h before sunrise (dark) in transgenic lines F, H and SFH compared to C. All transgenic lines were shown to have consistently higher levels of sucrose, with these levels being significantly higher than C in F and SF lines. SF lines were also shown to have a significantly higher amount of glucose (Figure 7) compared to C, although other lines were consistently elevated but not significantly so.

### Impact of light intensity on biomass and seed yield

**Figure 8:**
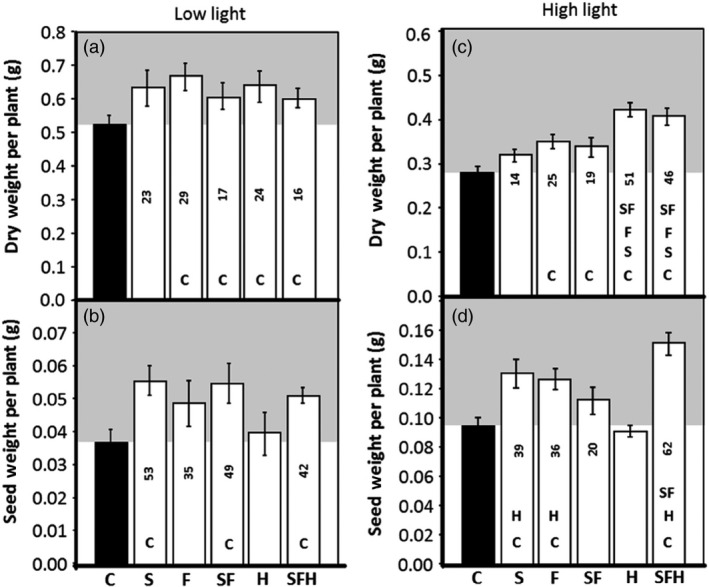
GDC‐H and GDC‐H with SBPase and FBPA overexpression in Arabidopsis differentially impact biomass and seed yield. (a, c) Dry weight and (b, d) seed weight were determined at seed harvest. C and transgenic plants were grown in controlled environment conditions at either 130 μmol/m^2^/s, 8‐h light/16‐h dark cycle (a., b.) or 390 μmol/m^2^/s, 8‐h light/16‐h dark cycle (c., d.). Lines over‐expressing SBPase (S), FBPA (F), GDC‐H protein (H), SBPase and FBPA (SF) and SBPase, FBPA and GDC‐H (SFH) are represented. The data were obtained using 10–17 individual plants from two (H), three (S, F, SF) or four (SFH) independent transgenic lines (2 H lines. See Timm *et al*., 2012) compared to 12–13 C. Columns represent mean values and standard errors are displayed. Significant differences between lines (*P* < 0.05) are represented as capital letters indicating whether each specific line is significantly different from another. Numbers indicate % increases over C. Seed weights per plant and full statistical evaluation between groups can be seen in Figure S10.

A subset of plants was allowed to seed in either low or high light, and final vegetative biomass and seed yield determined per plant. In low‐light grown plants, the final vegetative biomass was higher in all of the transgenic lines compared to C; however, no significant differences were observed between the different transgenic lines (Figure 8a). Furthermore, seed yield was increased by 35%–53% in transgenic lines S, SF and SFH (Figure 8b). Interestingly, no increase in seed yield was observed in the H plants.

We next compared the impact of growth of plants in high light to explore further the potential positive impact of these transgenic manipulations on growth. In high‐light grown plants, an increase in vegetative biomass from 14% to 51% was observed (Figures 8c and S9). Notably, the H and SFH plant produced significantly more vegetative biomass than the S, F, SF or C plants. Furthermore, seed yield in high‐light‐grown plants was increased by 39%–62% in transgenic lines S, F, SF and SFH, when compared to C (Figure 8d). Although the highest increase in seed yield was observed in lines SFH in high light, no increase in seed yield was observed in the H plants in high‐light‐grown plants. The seed yield for individual plants can be seen in Figure S10.

## Discussion

In this study, we have shown that simultaneously increasing the levels of two enzymes of the CB cycle, SBPase and FBPA, and the H protein of the glycine decarboxylase enzyme of the photorespiratory pathway in the same plant, resulted in a substantial and significant increase in both vegetative biomass and seed yield of Arabidopsis grown in controlled environment conditions. An increase in both biomass and yield was also observed in plants overexpressing SBPase or FBPA alone or in combination. However, although overexpression of GDC‐H alone resulted in an increase in vegetative biomass, no increase in seed yield was evident in these plants, grown in either low‐ or high‐light conditions. The reasons for this differential effect on seed yield have not yet been elucidated but may be due to changes in carbon status brought about by altered source/sink allocation which is supported by changes to starch and sucrose levels at the end of the night period in some of these lines. Higher levels of sucrose (and fructose, maltose) have also been observed in GDC‐L over‐expressers (Timm *et al*., 2015), and the over‐expression of GDC‐L enhances the metabolic capacity of photorespiration and is believed to alter the carbon flow through the TCA cycle (Timm *et al*., 2015).

It was shown in earlier studies that over‐expression of FBPA or SBPase alone in tobacco results in a stimulation of photosynthesis and biomass, with the greatest effect being seen in plants grown under elevated CO_2_ (Lefebvre *et al*., 2005; Rosenthal *et al*., 2011; Uematsu *et al*., 2012). Furthermore, when FBPA was over‐expressed in combination with SBPase in tobacco, this led to a cumulative increase in biomass in plants grown in ambient CO_2_ under greenhouse conditions (Simkin *et al*., 2015). Interestingly, in this current study, we have shown that in Arabidopsis that the over‐expression of FBPA alone, under current atmospheric CO_2_ levels, results in a stimulation of photosynthesis and increase in biomass on a similar level to that observed by over‐expression of SBPase alone. However, contrary to the results obtained in tobacco, the co‐expression of SBPase and FBPA in Arabidopsis did not lead to a further significant increase in either leaf area or biomass when compared to plants independently expressing SBPase (resulting in higher endogenous FBPA activity) or FBPA. This lack of differential effect of the co‐overexpression of SBPase and FBPA in this study can likely be explained by the fact that over‐expression of SBPase in Arabidopsis also led to a small but significant increase in endogenous FBPA protein levels and activity (25%–36%). Given that no increase in SBPase was present in the FBPA plants, this would suggest that in Arabidopsis, the stimulation in the SBPase, FBPA and the SF over‐expression lines may be due to increased FBPA activity. This is in contrast to tobacco where over‐expression of SBPase alone led to an increase in biomass and no increases in endogenous FBPA activity, highlighting the differences between species (Lefebvre *et al*., 2005; Rosenthal *et al*., 2011; Simkin *et al*., 2015).

Detailed analysis of a range of photosynthesis parameters revealed a similar increase in *A*_sat_ at low [O_2_] for all of the transgenic lines studied. The most significant increase was observed in SF lines which showed a 44% increase over control plants, with the lowest increase of 19% being observed in the H plants. An evaluation of the electron transport rates at low [O_2_] in a subset of these plants showed that lines over‐expressing GDC‐H (both H and SFH) displayed higher photosynthetic electron transport rates compared to C and plants over‐expressing SBPase and FBPA (SF). These results are in keeping with the previous study by Timm *et al*. (2012). All of the transgenic lines analysed here showed an increase in photosynthesis under high light and ambient CO_2_ conditions. However, under high light and saturated levels of CO_2_ the rate of assimilation in the H plants was similar to C, and this is in contrast to all other transgenic lines. This observation is in keeping with the proposal that over‐expression of the H protein stimulates the flow of carbon through the photorespiratory pathway, thereby reducing steady‐state levels of inhibitory photorespiratory metabolites, which in turn stimulates flux through the CB cycle. Whilst this hypothesis is supported by metabolite data and the observation that growth of GDC‐H plants is not stimulated when these plants are grown in elevated CO_2_ conditions (Timm *et al*., 2012), the exact mechanism of such feedback from photorespiration to the CB cycle is not yet known. The effect of these manipulations on photosynthesis was also determined at the growth light intensity where small differences in *A* are observed even at light levels as low as 150 μmol/m^2^/s. This together with the increased leaf area observed at early stages in development provides evidence that the small differences in photosynthesis lead to an increase in leaf area. The cumulative impact of this over time results in increased biomass and yield.

## Conclusion

In this proof‐of‐concept study in Arabidopsis, we have demonstrated that the simultaneous over‐expression of two CB cycle enzymes leads to an increase in photosynthesis and an increase in overall biomass and seed yield. We also show that when the transgenic SF lines were crossed with GDC‐H over‐expressing plants (Timm *et al*., 2012), the combined effects of these three transgenes (SFH) resulted in a cumulative impact on biomass (+71%) which was significantly higher than H (+50%) and SF (+41%) under low light. Importantly, the work here also allowed a parallel comparative analysis between the different manipulations under different environmental conditions.

Although it is still necessary to address the importance of these manipulations in crop species and also under field conditions, this study provides additional evidence that multigene manipulation of photosynthesis and photorespiration can form an important tool to improve crop yield. These results also provide new information indicating that it will be necessary to tailor the targets for manipulation for different crops and for either biomass or seed yield.

## Materials and methods

### Generation of pGW photosynthetic tissue‐specific destination vector pGWPTS1

pGWB1 (Nakagawa *et al*., 2007: AB289764) was cut with HindIII at 37 °C. Following purification, digested vectors were treated with alkaline phosphatase (BioLabs, UK) for 60 min at 37 °C. The rbcS2B (1150 bp; *At5 g38420*) promoter was amplified with primers Pr_rbcS2B_F_HindIII (5′CACCaagcttATgACATCATAgCAAgCAAggACACg'3) and Pr_rbcS2B_R_HindIII (5′CTGAGAaagcttTACTTCTTCTTgTTgTTTCTCTTCTTC'3). The amplicon was digested with HindIII and cloned into the corresponding site of pGWB1 to make vector pGWPTS1 (Figure S1a).

Constructs were generated using Gateway cloning technology and vector pGWPTS1. All transgenes were under the control of the rbcS2B (1150 bp; *At5 g38420*) promoter. Full details of PTS1‐SB, PTS1‐FB and PTS1‐SBFB construct assembly can be seen in the supplementary data. Construct maps are shown in Figure S1b–d.

### Generation of transgenic plants

The recombinant plasmids PTS1‐SB, PTS1‐FB and PTS1‐SBFB were introduced into wild‐type Arabidopsis by floral dipping (Clough and Bent, 1998) using *Agrobacterium tumefaciens* GV3101. Positive transformants were regenerated on MS medium containing kanamycin (50 mg/L) and hygromycin (20 mg/L). Kanamycin‐/hygromycin‐resistant primary transformants (T1 generation) with established root systems were transferred to soil and allowed to self‐fertilize. Plants over‐expressing SBPase, FBPA and the GDC‐H protein were generated by floral inoculation of two SBPase + FBPA lines (SF6 and SF12) with the pollen from two GDC‐H protein over‐expressing lines (*Fp*H17 and *Fp*H18) provided by Timm *et al*. (2012). Lines *Fp*H17 and 18 were originally generated by floral dipping and over‐expressing the *Flaveria pringlei* GDC‐H protein (Kopriva and Bauwe, 1995) under the control of the leaf‐specific and light‐regulated *Solanum tuberosum ST‐LS1* promoter (Stockhaus *et al*., 1989). Following initial characterization of generated lines, three lines for SBPase (S3, S8, S12), FBPA (F6, F9, F11) and SF (SF6, SF7, SF12) were selected for further study from all lines generated.

### Plant growth conditions

Wild‐type T2 Arabidopsis plants resulting from self‐fertilization of transgenic plants were germinated in sterile agar medium containing Murashige and Skoog salts (plus kanamycin 50 mg/L for the transformants) and grown to seed in soil (Levington F2, Fisons, Ipswich, UK). Lines of interest were identified by immunoblot and qPCR. For experimental study, T3 progeny seeds from selected lines were germinated on soil in controlled environment chambers at an irradiance of 130 μmol photons/m^2^/s, 22 °C, relative humidity of 60%, in an 8‐h/16‐h square‐wave photoperiod. Plants were sown randomly, and trays rotated daily. Four leaf discs (0.6 cm diameter) from two individual leaves, for the analysis of SBPase and FBPA activities, were taken and immediately plunged into liquid N_2_, and stored at −80 °C. Leaf areas were calculated using standard photography and ImageJ software (imagej.nih.gov/ij). Wild‐type plants and null segregants (azygous) used in this study were initially evaluated independently. However, once it was determined that no significant difference were observed between these two groups (see figures and supplementary figures), wild‐type plants and null segregants were combined (null segregants from the transgenic lines verified by PCR for nonintegration of the transgene) and used as a combined ‘control’ group (C).

### Protein extraction and immunoblotting

Leaf discs sampled as described above were ground in liquid nitrogen. Total protein was extracted in extraction buffer (50 mm 4‐(2‐hydroxyethyl)piperazine‐1‐ethanesulphonic acid (HEPES) pH 8.2, 5 mm MgCl2, 1 mm ethylenediaminetetraacetic acid tetrasodium salt (EDTA), 10% glycerol, 0.1% Triton X‐100, 2 mm benzamidine, 2 mm aminocaproic acid, 0.5 mm phenylmethanesulphonyl fluoride (PMSF) and 10 mm DTT), and the insoluble material was removed by centrifugation at 14 000 *g* for 10 min (4 °C) and protein quantification determined (Harrison *et al*., 1998). Samples were loaded on an equal protein basis, separated using 12% (w/v) SDS‐PAGE, transferred to polyvinylidene difluoride membrane and probed using antibodies raised against SBPase, FBPA and the GDC‐H protein (Timm *et al*., 2012). Proteins were detected using horseradish peroxidase conjugated to the secondary antibody and ECL chemiluminescence detection reagent (Amersham, Buckinghamshire, UK). SBPase antibodies are previously characterized in Lefebvre *et al*. (2005), and FBPA antibodies were raised against a peptide from a conserved region of the protein [C]‐ASIGLENTEANRQAYR‐amide, Cambridge Research Biochemicals, Cleveland, UK (Simkin *et al*., 2015). In addition to the aforementioned antibodies, samples were probed using antibodies raised against the phosphoribulokinase (AS09464), ssAGPase (AS111739), purchased from Agrisera (via Newmarket Scientific, UK) and FBPase (see Lefebvre *et al*., 2005), transketolase (Henkes *et al*., 2001) and Rubisco (Foyer *et al*., 1993).

### Determination of SBPase activity by phosphate release

SBPase activity was determined by phosphate release. Immediately after photosynthesis measurement, leaf discs were isolated from the same leaves and frozen in liquid nitrogen. For analysis, leaf discs were ground to a fine powder in liquid nitrogen in extraction buffer (50 mm HEPES, pH8.2; 5 mm MgCl_2_; 1 mm EDTA; 1 mm EGTA; 10% glycerol; 0.1% Triton X‐100; 2 mm benzamidine; 2 mm aminocaproic acid; 0.5 mm phenylmethylsulphonylfluoride; 10 mm dithiothreitol), and the resulting solution was centrifuged 1 min at 14 000 *g*, 4 °C. The resulting supernatant was desalted through an NAP‐10 column (Amersham) and eluted, aliquoted and stored in liquid nitrogen. For the assay, the reaction was started by adding 20 μL of extract to 80 μL of assay buffer (50 mm Tris, pH 8.2; 15 mm MgCl_2_; 1.5 mm EDTA; 10 mm dithiothreitol; 2 mm SBP) and incubated at 25 °C for 30 min as described previously (Simkin *et al*., 2015). The reaction was stopped by the addition of 50 μL of 1 m perchloric acid and centrifuged for 10 min at 14 000 *g*, 4 °C. Samples (30 μL) and standards (30 μL, 0.125–4 nmol PO^3−^_4_) in triplicate were incubated 30 min at room temperature following the addition of 300 μL of Biomol Green (Affiniti Research Products, Exeter, UK), and the A_620_ was measured using a microplate reader (VERSAmax, Molecular Devices, Sunnyvale, CA).

### Determination of FBPA activity

Desalted protein extracts, as described above, were evaluated for FBPA activity as described previously (Haake *et al*., 1998).

### Chlorophyll fluorescence imaging

Measurements were performed on 2‐week‐old Arabidopsis seedlings that had been grown in a controlled environment chamber providing 130 μmol/mol^2^/s PPFD and ambient CO_2_. Chlorophyll fluorescence parameters were obtained using a chlorophyll fluorescence (CF) imaging system (Technologica, Colchester, UK; Barbagallo *et al*., 2003; Baker and Rosenqvist, 2004). The operating efficiency of photosystem two (PSII) photochemistry, *F*_q_'/*F*_m_', was calculated from the measurements of steady‐state fluorescence in the light (*F*') and maximum fluorescence in the light (*F*_m_') since *F*_q_'/*F*_m_' = (*F*_m_' − *F*')/*F*_m_'. Images of *F*' were taken when fluorescence was stable at 130 μmol/m^2^/s PPFD, whilst images of maximum fluorescence were obtained after a saturating 600 ms pulse of 6200 μmol/m^2^/s PPFD (Baker *et al*., 2001; Oxborough and Baker, 1997). Parallel measurements of plants grown in high light (390 μmol/mol^2^/s PPFD and ambient CO_2_) were also performed on 2‐week‐old Arabidopsis (Supporting Information).

### Gas exchange measurements

The response of net photosynthesis (*A*) to intracellular CO_2_ (C_i_) was measured using a portable gas exchange system (CIRAS‐1, PP Systems Ltd, Ayrshire, UK). Leaves were illuminated with an integral red‐blue LED light source (PP systems Ltd) attached to the gas exchange system, and light levels were maintained at saturating photosynthetic photon flux density (PPFD) of 1000 μmol/m^2^/s for the duration of the A/C_i_ response curve. Measurements of *A* were made at ambient CO_2_ concentration (C_a_) at 400 μmol/mol, before C_a_ was decreased to 300, 200, 150, 100 and 50 μmol/mol before returning to the initial value and increased to 500, 600, 700, 800, 900, 1000, 1100 and 1200 μmol/mol. Leaf temperature and vapour pressure deficit (VPD) were maintained at 22 °C and 1 ± 0.2 kPa, respectively. The maximum rates of Rubisco‐ (*Vc*_max_) and the maximum rate of electron transport for RuBP regeneration (*J*_max_) were determined and standardized to a leaf temperature of 25 °C based on equations from Bernacchi *et al*. (2001) and McMurtrie and Wang (1993), respectively.

### Photosynthetic light response curves

A/Q response curves were measured using a CIRAS‐1 portable gas exchange system (PP Systems (CIRAS‐1, PP Systems Ltd). Cuvette conditions were maintained at a leaf temperature of 22 °C, relative humidity of 50%–60% and ambient growth CO_2_ concentration (400 mmol/mol for plants grown in ambient conditions). Leaves were initially stabilized at saturating irradiance 1000 μmol/m^2^/s, after which *A* and *g*_s_ were measured at the following PPFD levels: 0, 50, 100, 150, 200, 250, 300, 350, 400, 500, 600, 800 and 1000 μmol/m^2^/s. Measurements were recorded after *A* reached a new steady state (1–2 min) and before stomatal conductance (*g*_s_) changed to the new light levels. A/Q analyses were performed at 21% and 2% O_2_.

### Determination of sucrose and starch

Carbohydrates and starch were extracted from 20 mg leaf tissue, and samples were collected at two time points, 1 h before dawn (15 h into the dark period) and 1 h before sunset (7 h into the light period). Four leaf discs collected from two different leaves were ground in liquid nitrogen, and 20 mg/FW of tissue was incubated in 80% (v/v) ethanol for 20 min at 80 °C and then repeated three times with ethanol 80% (v/v) at 80 °C. The resulting solid pellet and pooled ethanol samples were freeze‐dried. Suc was measured from the extracts in ethanol using an enzyme‐based protocol (Stitt *et al*., 1989), and the starch contents were estimated from the ethanol‐insoluble pellet according to Stitt *et al*. (1978), with the exception that the samples were boiled for 1 h and not autoclaved.

### Statistical analysis

All statistical analyses were performed by comparing ANOVA, using Sys‐stat, University of Essex, UK. The differences between means were tested using the *post hoc* Tukey test (SPSS, Chicago, IL).

## Author contributions

C.A.R. conceived this project, provided the funding and led the supervision of this research with input from T.L. A.J.S generated transgenic plants and performed molecular, biochemical and plant phenotypic analysis. L.R.H and P.E.L contributed to the generation and analysis of the transgenic plants. A.J.S and P.A.D carried out data analysis on their respective contributions. S.T and H.B generated and provided glycine decarboxylase over‐expressing lines used for crosses. The manuscript was drafted by A.J.S and finalised by C.A.R. All authors reviewed and commented on the final manuscript.

## Conflict of interest

The authors declare no conflict of interest.

## Supporting information


**Figure S1** Schematic representation of the (a) vector pGWPTS1, (b) *A. thaliana SBPase* (PTS1SB) over‐expression construct, and the (c) *A. thaliana FBPA* (PTS1‐FB) over‐expression construct, (d) shows the structure of a duel construct for the expression of both *SBPase* and *FBPA* (PTS1‐SBFB).
**Figure S2** (a) Complete data set for SBPase enzyme assays in plants analysed. (b) Complete data set for FBP aldolase enzyme assays in plants analysed.
**Figure S3** Molecular and biochemical analysis of the transgenic plants overexpressing SBPase (S), FBPA (F) or both (SF).
**Figure S4** (a) The operating efficiency of PSII photochemistry of C and transgenic plants at 600 μmol/m^2^/s light. Capacity determined using chlorophyll fluorescence imaging. (b) the maximum carboxylation activity of Rubisco and (c) *J*_max_ were derived from *A*/*C*_*i*_ response curves (Figure 4).
**Figure S5** Photosynthesis carbon fixation rates determined as a function of light intensity in developing leaves.
**Figure S6**. Complete data set for all transgenic lines evaluated. (a) leaf area at 15 days, (b) Leaf area at 20 days (c) Leaf area at 25 days.
**Figure S7** Growth analysis of the transgenic and control plants grown in low light.
**Figure S8** Leaf number in control and transgenic lines.
**Figure S9** Complete data set for leaf area of all transgenic lines evaluated at high light (390 μmol/m^2^/s).
**Figure S10** Complete data set for seed yield (g) from all transgenic lines evaluated in (a) low light (130 μmol/m^2^/s) and (b) high light (390 μmol/m^2^/s).
