# Supplementary material for: Simultaneous stimulation of sedoheptulose 1,7‐bisphosphatase, fructose 1,6‐bisphophate aldolase and the photorespiratory glycine decarboxylase‐H protein increases CO 2 assimilation, vegetative biomass and seed yield in Arabidopsis
Source: Plant Biotechnol J. 2017 Mar 21;15(7):805–16. doi: 10.1111/pbi.12676 (PMC5466442; doi:10.1111/pbi.12676)
Supplement: Supplementary file 1 — Figure S1 Schematic representation of the (a) vector pGWPTS1, (b) A. thaliana SBPase (PTS1SB) over‐expression construct, and the (c) A. thaliana FBPA (PTS1‐FB) over‐expression construct, (d) shows the structure of a duel construct for the expression of both SBPase and FBPA (PTS1‐SBFB). Figure S2 (a) Complete data set for SBPase enzyme assays in plants analysed. (b) Complete data set for FBP aldolase enzyme assays in plants analysed. Figure S3 Molecular and biochemical analysis of the transgenic plants overexpressing SBPase (S), FBPA (F) or both (SF). Figure S4 (a) The operating efficiency of PSII photochemistry of C and transgenic plants at 600 μmol/m2/s light. Capacity determined using chlorophyll fluorescence imaging. (b) the maximum carboxylation activity of Rubisco and (c) J max were derived from A/C i response curves (Figure 4). Figure S5 Photosynthesis carbon fixation rates determined as a function of light intensity in developing leaves. Figure S6. Complete data set for all transgenic lines evaluated. (a) leaf area at 15 days, (b) Leaf area at 20 days (c) Leaf area at 25 days. Figure S7 Growth analysis of the transgenic and control plants grown in low light. Figure S8 Leaf number in control and transgenic lines. Figure S9 Complete data set for leaf area of all transgenic lines evaluated at high light (390 μmol/m2/s). Figure S10 Complete data set for seed yield (g) from all transgenic lines evaluated in (a) low light (130 μmol/m2/s) and (b) high light (390 μmol/m2/s). [file PBI-15-805-s001.docx]

*Supplementary Information*

**
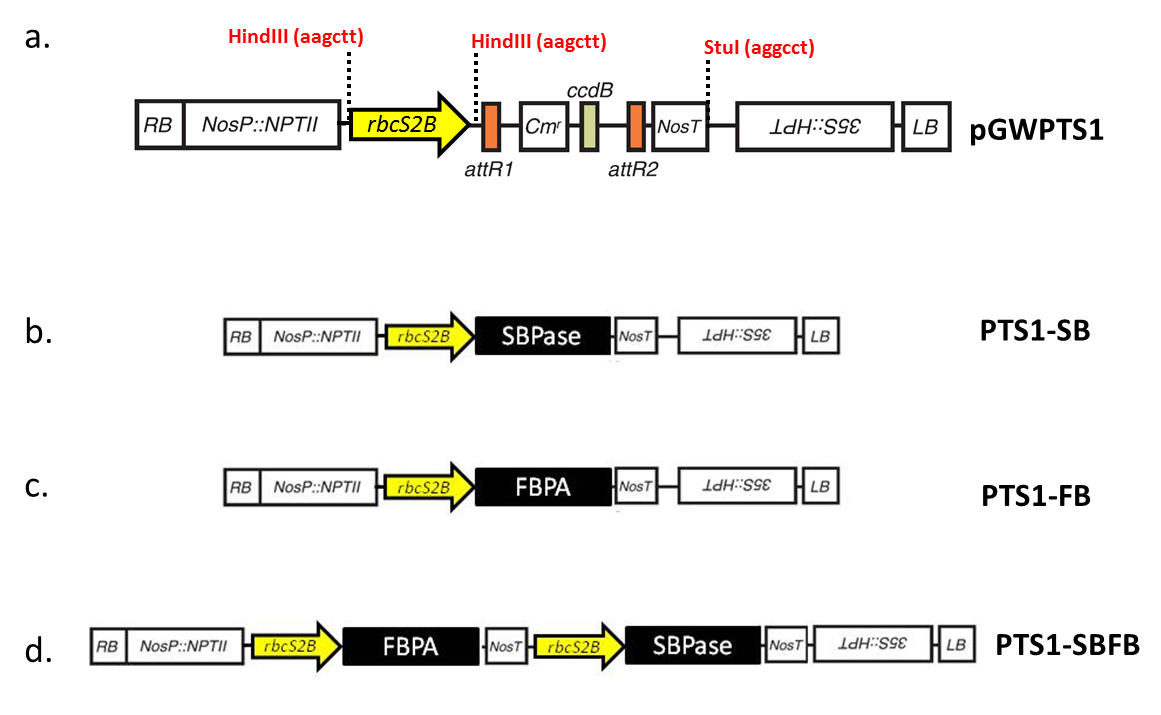
**

**Figure S1** Schematic representation of the (a) vector pGWPTS1, (b) *A. thaliana SBPase* (PTS1SB) over-expression construct, and the (c) *A. thaliana FBPA* (PTS1-FB) over-expression construct, (d) shows the structure of a duel construct for the expression of both *SBPase* and *FBPA* (PTS1-SBFB). RB, T-DNA right border; Pnos, nopaline synthase promoter; NTP II, neomycin phosphotransferase gene; Tnos, nopaline synthase terminator; P35S, rbcS2B promoter (1150bp; *At5g38420*). Constructs were used to transform wild Arabidopsis (Col-0)


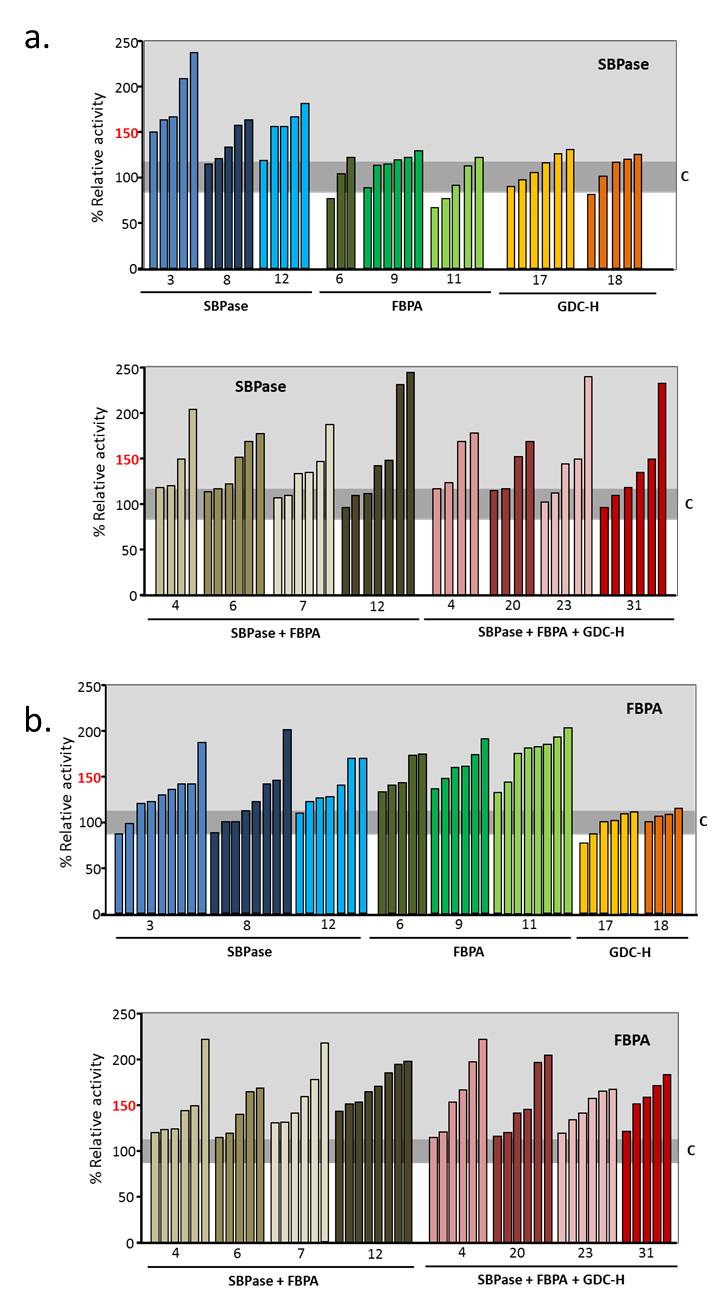


**Figure S2** (a) Complete data set for SBPase enzyme assays in plants analysed. (b) Complete data set for FBP aldolase enzyme assays in plants analysed. The results are represented as a percentage (%) of total activity for SBPase (6.7 μmol m^-2^ s^-1^) and FBPaldolase (22 μmol m^-2^ s^-1^) determined in wild type control (C). Results are displayed for each individual plant. Lines over-expressing SBPase (S), FBPA (F), GDC-H protein (H), SBPase and FBPA (SF), and SBPase, FBPA and GDC-H (SFH) are represented. C standard error is indicated by the dark grey bar. See Figure 1.


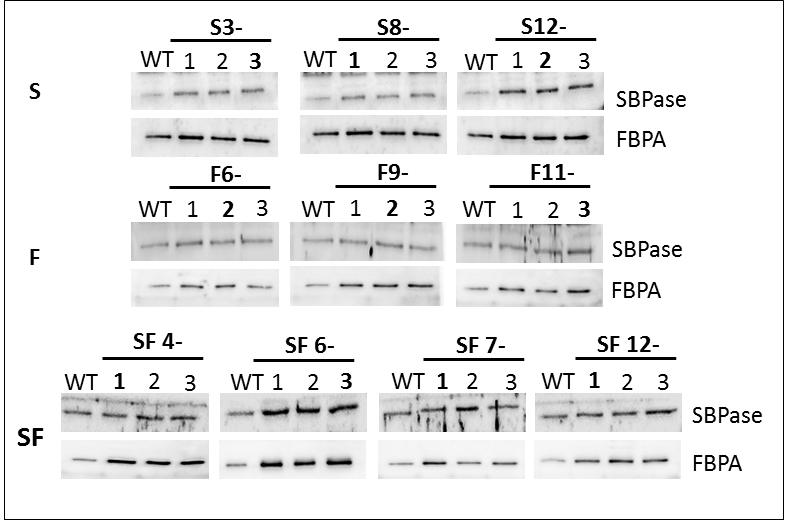


**Figure S3** Molecular and biochemical analysis of the transgenic plants overexpressing SBPase (S), FBPA (F) or both (SF). Immunoblot analysis (SBPase, FBPA) of protein extracts from selected lines compared to WT. See Figure 1.


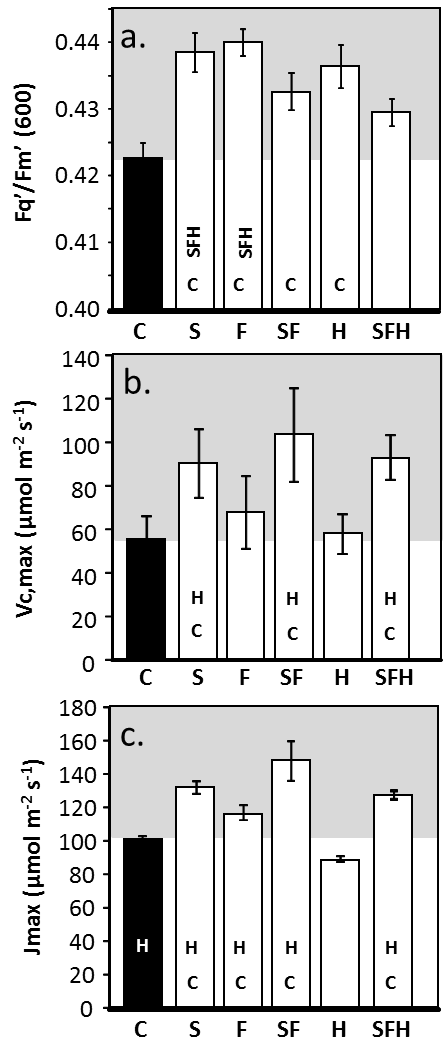


**Figure S4** (a) The operating efficiency of PSII photochemistry of C and transgenic plants at 600 μmol m^-2^ s^-1^ light. Capacity determined using chlorophyll fluorescence imaging. (b) the maximum carboxylation activity of Rubisco and (c) *J*_max_ were derived from *A*/*C_i_* response curves (see Figure 4). Plants were grown in controlled environment conditions with a light intensity 130 µmol m^-2^ s^-1^, 8 h light/16 h dark cycle for 15 d. Significant differences (p<0.05) are represented as capital letters indicating if each specific line is significantly different from another.


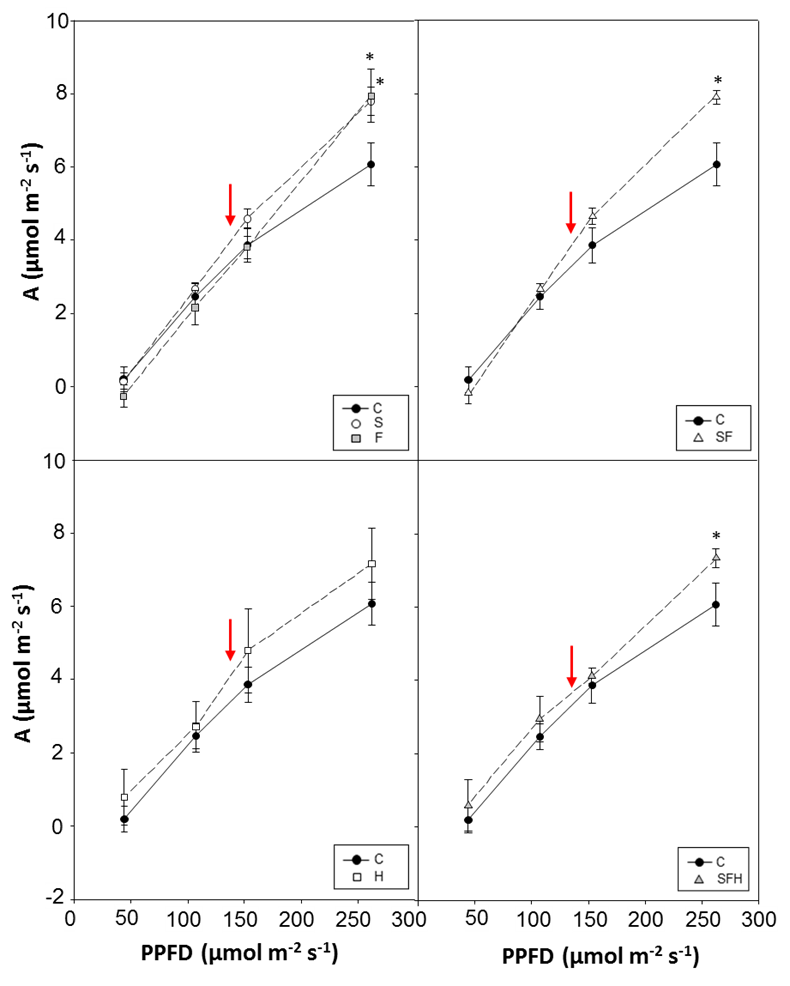


**Figure S5** Photosynthesis carbon fixation rates determined as a function of light intensity in developing leaves. Control (C) and transgenic plants were grown in controlled environment conditions with a light intensity 130 µmol m^-2^ s^-1^, 8h light/16h dark cycle for four weeks. Red arrow indicates growth light intensity. Lines over-expressing SBPase (S), FBPA (F), GDC-H protein (H), SBPase and FBPA (SF), and SBPase, FBPA and GDC-H (SFH) are represented. Significant differences (* p<0.05) are represented. Results are based on 4 to 7 plants per line. See Figure 4.


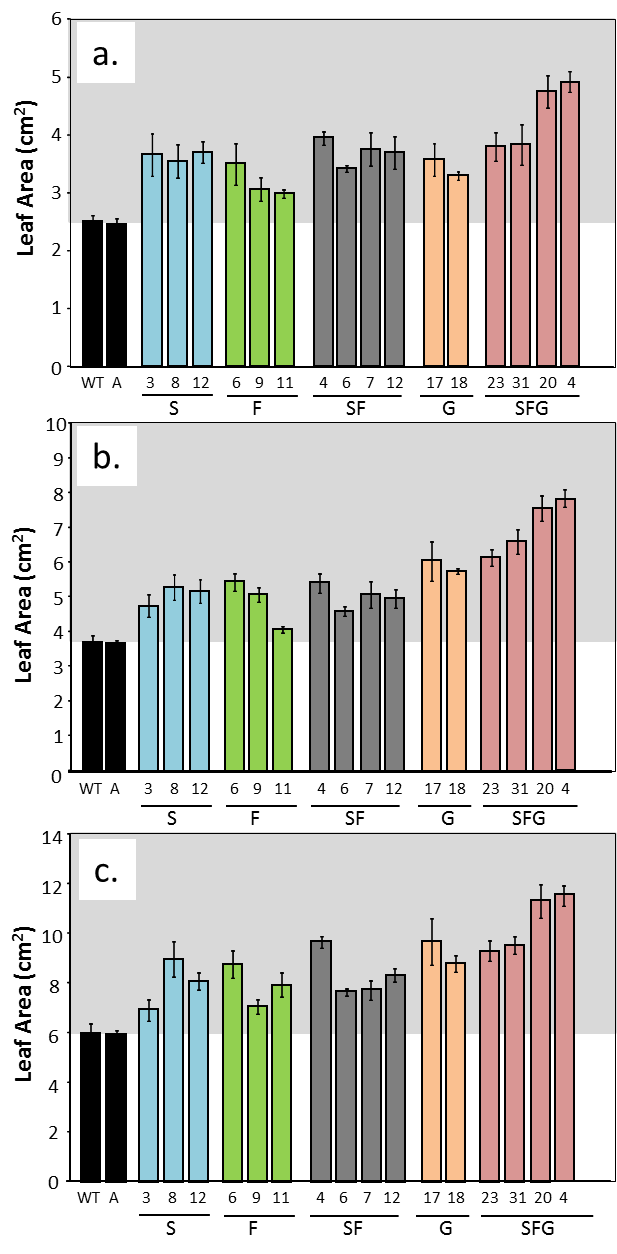


**Figure S6** Complete data set for all transgenic lines evaluated. (a) leaf area at 15 days, (b) Leaf area at 20 days (c) Leaf area at 25 days. Lines over-expressing SBPase (S), FBPA (F), GDC-H (H), SBPase and FBPA (SF), SBPase, FBPA and GDC-H (SFH) are represented. Each column represents 4 to 5 plants. AZY (A) represents WT lines segregated from transgenic lines following transformation. Plants were grown at 130 µmol m^-2^ s^-1^ light intensity in short days (8h/16h days). See Figure 6.


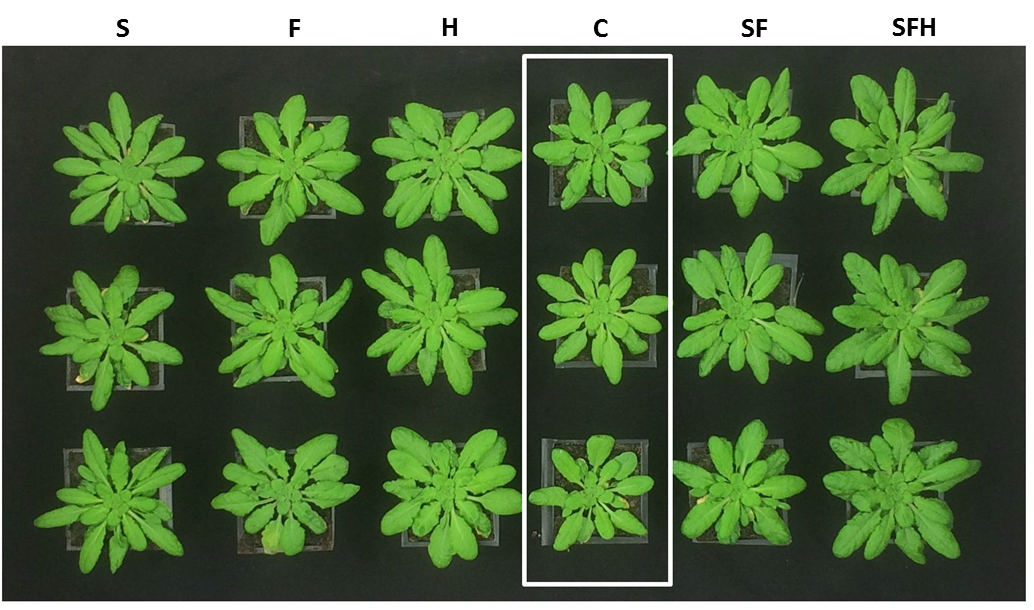


**Figure S7** Growth analysis of the transgenic and control plants grown in low light. Plants were grown for 38 days at 130 µmol m^-2^ s^-1^ light intensity in short days (8h/16h days). Lines over-expressing a single transgene, SBPase (S), FBPA (F), GDC-H protein (H), two transgenes, SBPase and FBPA (SF), or three transgenes, SBPase, FBPA and GDC-H (SFH) are shown. See Figure 6.


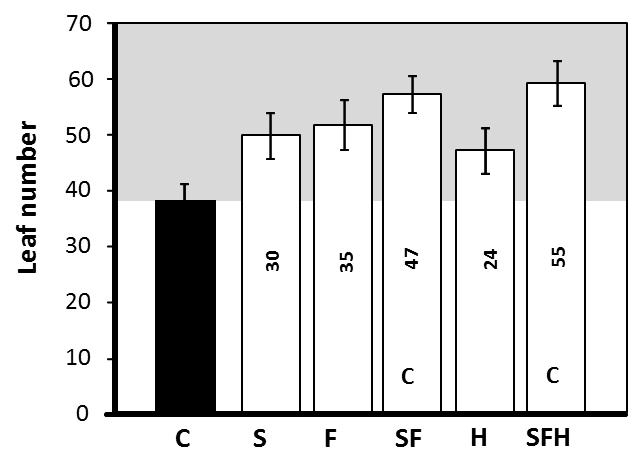


**Figure S8** Leaf number in Control and transgenic lines. Plants were grown for 38 days at 130 µmol m^-2^ s^-1^ light intensity in short days (8h/16h days). Lines over-expressing SBPase (S), FBPA (F), GDC-H (H), SBPase and FBPA (SF), SBPase, FBPA and GDC-H (SFH) are represented. Plants were grown at 130 µmol m^-2^ s^-1^ light intensity in short days (8h/16h days). Significant differences (p<0.05) are represented as capital letters indicating if each specific line is significantly different from another. See Figure 6.b


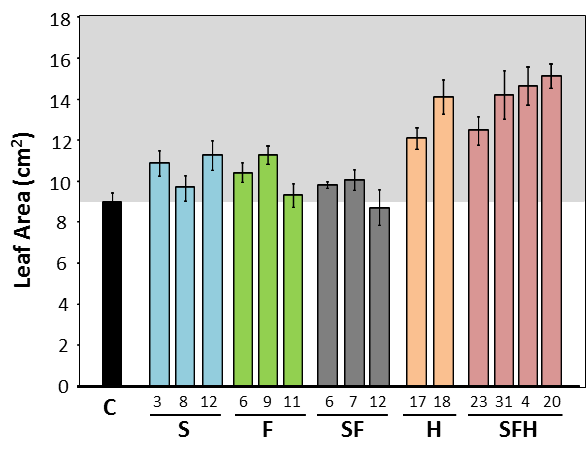


**Figure S9** Complete data set for Leaf area of all transgenic lines evaluated at high light (390 µmol m^-2^ s^-1^). Leaf area following 15 d growth in high light, Lines over-expressing SBPase (S), FBPA (F), GDC-H (H), SBPase and FBPA (SF), SBPase, FBPA and GDC-H (SFH) are represented compared to controls (C). Plants were grown at 130 µmol m^-2^ s^-1^ light intensity in short days (8 h/16 h). Each column represents 4 to 5 plants. See Figure 8.


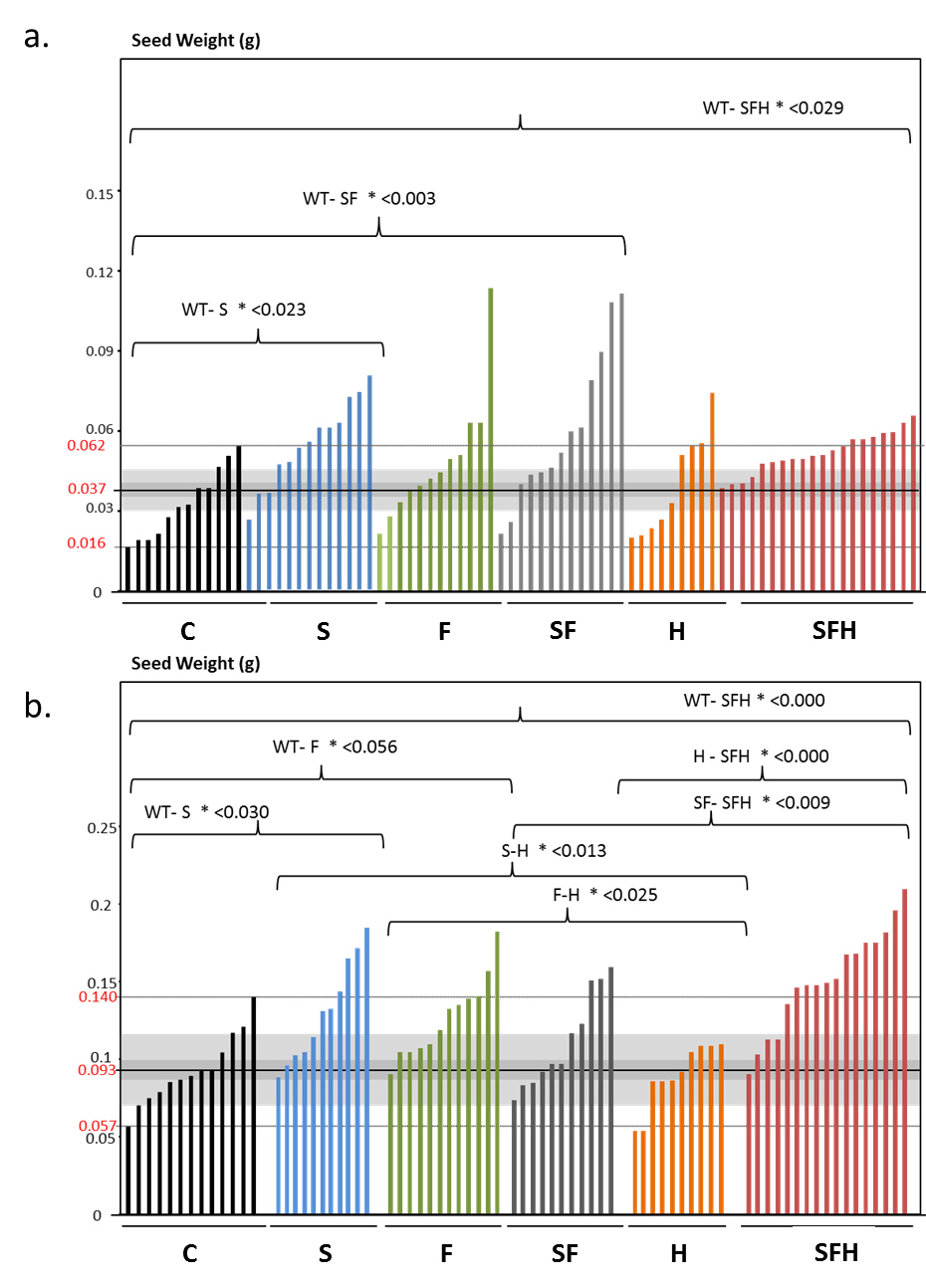


**Figure S10** Complete data set for seed yield (g) from all transgenic lines evaluated in (a) low light (130 µmol m^-2^ s^-1^) and (b) high light (390 µmol m^-2^ s^-1^). Seed weight (g) per plant is indicated. Lines over-expressing SBPase (S), FBPA (F), GDC-H (H), SBPase and FBPA (SF), SBPase, FBPA and GDC-H (SFH) are represented compared to controls (C). Plants were grown at 130 µmol m^-2^ s^-1^ light intensity in short days (8h/16h days). Dark grey box represents standard error and pale grey standard deviation. Statistical differences between lines are shown. See Figure 8.
